# Closing the gap between knowledge and application: a call for consensus, practice-oriented sport nutrition assessment tools among youth athletes

**DOI:** 10.3389/fnut.2026.1924608

**Published:** 2026-09-08

**Authors:** Alysha L. Deslippe, Floris C. Wardenaar

**Affiliations:** 1 Department of Health, Kinesiology and Applied Physiology, Concordia University, Montreal, QC, Canada; 2 College of Health Solutions, Arizonia State University, Phoenix, AZ, United States

**Keywords:** adolescents, athlete, dietary intake, dietary quality, knowledge, macro- and micronutrients

## Introduction

Nutrition plays a critical role in an athletes' health and performance (1–3). In the literature, an athlete's knowledge of their nutrient needs for sport (i.e., sport nutrition knowledge) has been found to be a targetable strategy to effectively optimize health and performance (4). Despite this, there is no consensus on what knowledge an athlete needs, and how it should be assessed. Instead, researchers have utilized diverse measurement tools in different contexts that vary substantially (5–10). Given the energy demands sport places on an athletes' body, knowledge of adequate energy (or caloric) needs is consistently reported as an important knowledge domain for all athletes regardless of their age, sex, training load and other socio-cultural factors that impact fueling (1–3). Assessment of sports nutrition knowledge has further assessed knowledge domains surrounding macro- and micro-nutrient needs, hydration, safe supplement use and timing of nutrient intake (1–3). In light of this diversity, we believe that frequently used tools that measure sport nutrition knowledge are not leveraged to their full potential to favorably impact youth athletes given their developmental stage.

## Youth athletes are unique

Youth undergoing puberty (~11–18 years) experience rapid physical growth and cognitive development. Typically, this growth result in higher nutrient needs compared to earlier childhood (e.g., energy needs) and adulthood (e.g., calcium), though some nutrients are needed in lower absolute quantities (11). Youth often begin to have greater dietary independence and consume more meals away from their caregivers (12). Thus, we believe that it is critical youth athletes are aware of their changing needs and prepared to meet them independently. An important example of this can be seen with the micro-nutrient iron; Menstruating female athletes have increased iron needs compared to their male counterparts, regardless of their training load (11). Despite the well-established clinical importance of iron (whether from foods or supplements) for an athlete (1), assessment of youth female athletes' knowledge of their increased iron needs is rare. Further, suitable validated research tools, aside from measurement of clinical biomarkers (e.g., through a blood test), are only available for a limited number of nutrients and not routinely provided as part of youth athletic programs. Instead, we see potential to leverage sports nutrition knowledge assessment tools within sports organizations (e.g., clubs and schools) as a strategy to help prepare youth athletes to manage their changing nutrient needs independently, including nutrients like iron.

## Frequently used assessment techniques

In research, sport nutrition knowledge has been assessed using pen and paper or digital surveys (5–10). A 2016 systematic review that assessment adult athletes, coaches and trainers sport nutrition knowledge reported a high degree of heterogeneity in the “*question types and formats across measures*” and what knowledge domains were assessed (13). Of the 36 studies, 35 asked about carbohydrates, 31 about protein, 32 about micronutrients, 21 about supplements, 23 about fluid intake, 17 about pre-competition fueling, and one about fueling during competition (13). Since 2016, two tools measuring sport nutrition knowledge have been developed for research and validated with youth athletes to our knowledge; The Abridged Sports Nutrition Knowledge Questionnaire (A-SNKQ) (14, 15) and nutrition knowledge for young and adult athletes (NUKYA) (16). Additional tools used to measure sport nutrition knowledge of youth athletes in research that have not been validated include a sport-specific measure (5, 6) and tools that ask about single nutrition domains (e.g., safe supplement use (8–10)). Regardless of the tool, most research reports the percentage of questions answered correctly to infer youth knowledge, with higher scores implying greater knowledge (5–10).

Regardless of the tool used sport nutrition knowledge among youth athletes is frequently reported as low (5–10). For example, a United States cross-sectional study reported less than 50% of surveyed youth answered A-SNKQ questions correctly (*n* = 29 males; *n* = 15 females; mean age 16.5 years) (7). Other literature has corroborated these findings utilizing different measures (6, 8–10). The totality of low scores reported across studies calls attention to a gap in youth athletes' sport nutrition knowledge. However, we offer a second explanation in that the way we conceptualize and measure sport nutrition knowledge among youth athletes may be part of the problem given our practical and clinical expertise working with youth athletes.

## Youth are not experts

Many tools measuring sport nutrition knowledge ask questions that require substantial expertise in nutrition and exercise physiology to answer. For example, a question from the A-SNKQ asking “*Do you think 1 medium banana has enough carbohydrate for recovery from intense exercise?”* with additional information to “*Assume the athlete weighs about 70 kg and has an important training session again tomorrow.”* To select one of the three responses (“*enough,” “not enough” or “not sure”*) an athlete needs knowledge of what a carbohydrate is, how many carbohydrates are in the average banana, carbohydrate's role in recovery, and what carbohydrate recommendations are based on body size and training intensity. Such concepts are expected of a trained sport nutrition expert (e.g., dietitian), but is it practical to expect youth athletes to correctly respond? We further wonder if assessing single food items (i.e., banana) is practically useful, given individual preferences and the broader family and food systems youth eat within (12, 17–19). Instead, we propose simplifying questions and creating space for athlete context. For example, asking “*If you compete for longer or are working very hard, what macro-nutrient should you consume more of?*” followed by “*Can you think of an example of a food rich in this nutrient that you already consume?”* In certain cases, it may be useful to further inquire about dose of a nutrient/food. We believe this altered approach may be more practical given an athletes' scope of knowledge.

## Memorizing clinical guidelines does not priorities practical fueling strategies

Many tools ask questions that require knowledge of clinical guidelines. For example, a true and false question from the NUKYA: “*Your athletic performance will decrease if you lose 2% of your body weight (for example, 1.5 kg if you weigh about 75 kg) due to water loss*.” While this 2% threshold may have clinical relevance, though debated among experts (20), it likely has limited practicality for an athlete to apply during training. We propose re-thinking the purpose behind questions to focus on behaviors an athlete can perform and/or clinical signs they can observe on their own. For example, “*True or False: An athlete experiencing dark urine, a headache and feeling faint during competition may be not drinking enough water during the day*”? Asking such a question may better assess an athletes' ability to understand dehydration in a way that they can use while competing. This approach may be particularly helpful in providing youth athletes with greater awareness of signs of harmful conditions like Low Energy Availability and Relative Energy Deficiency in Sport that can differ between male and female athletes (3).

## Knowledge is more than understanding nutrient needs

At present, majority of measurement tools emphasize questions that an athletes' knowledge of quantitative nutrient needs (e.g., amount of protein) based on body size and/or training load. Though important, this approach fails to assess youth athletes' broader knowledge of health literacy components (13) that have an established impact on dietary behaviors. Instead, including questions that assess broader skills and competencies that help an athlete make decisions given their socio-ecological context is warranted (21). For example, assessing youth athletes' ability to read a food label (intra-personal), navigate teammate conversations about food (inter-personal), identify venues with appropriate food options (community) and recognize how broader pressures, like gender-based body ideals (societal), can contradict what performance nutrition experts advise.

## Discussion

Sport nutrition is a complex topic as every athlete has unique needs (22) and context in which they make food decisions (17–19). To better understand how athletes' fuel their bodies, and identify gaps in nutrition programs and supports, we believe that sport nutrition knowledge assessment tools have potential to be leveraged. Importantly, the strategies outlined above are worthwhile to consider in our view. However, we acknowledge that other sport end-users, including other researchers, have valuable insights that may differ from ours and opinions are needed to effectively inform sport nutrition knowledge assessment. To move toward the creation and use of more practical tools, we call on the broader field to collaborate to define more clearly what practical sport nutrition knowledge entails and how it can be measured among youth.

### Consensus regarding practical sport nutrition knowledge

Given the lack of consensus on what knowledge domains are needed to confer sport nutrition knowledge (13), we believe an important first step hinges around clarifying what practical knowledge (or adequate knowledge to make practical decisions) entails. To parse out greater consensus, we call on the research community to collaborate with sport end-users to develop a Core Outcome Set (COS) (23) of practical sport nutrition knowledge among youth athletes.

A COS is a rigorous series of research steps developed by the COMET initiative to create a minimum set of outcomes that should be measured to assess sport nutrition knowledge in future efficacy and effectiveness trials (23). Notably, a COS builds off of the existing literature and integrates end-user feedback throughout. Starting with a systematic review, the COS process facilitates a comparison of the number of previously used items, nutrition domains captures, tool validity and reliability, and tailoring across different groups of athletes in prior tools (23). This extracted information is then used to create a list of items (including what is asked and how) intended to assess meaningful changes in knowledge to diverse sport end-users (23). This list is then discussed through methods like a Delphi study and followed by a consensus event to collaboratively refine what is assessed (23). Creating the COS would provide a minimum set of outcomes that can confer practical sport nutrition knowledge among youth athletes.

### Integrating broader knowledge competencies

To inform broader incorporation of skills (or competencies) that impact how youth eat, it would be useful for the field to collaboratively define a guiding framework of critical sport nutrition domains. To do this, we propose following procedures borrowed from the youth food literacy space where guiding frameworks such as the Food Literacy Competencies for Young Adults Framework have been established (24). This framework emphasizes developmental skills (or competencies) that youth need to successfully navigate complex food systems (e.g., reading food labels), develop positive food relationships (e.g., media literacy), and reduce their risk of negative health outcomes (e.g., knowledge of evidence-based recommendations) as they transition to adulthood (24). Development of a conceptual framework like this for youth athletes could provide substantial benefit in guiding the underlying questions asked within a tool measuring sport nutrition knowledge, and help infer more accurately that youth are prepared to apply evidence-based recommendations in their socio-ecological contexts.

### Assessing practical knowledge comes with a cost

Altering current measurement tools to assess what we argue is practical knowledge would likely create a higher burden for research teams. For example, altering questions to use more open-ended formats (e.g., asking an athlete to identify foods they already consume) would require a research team to check each individual response for accuracy. This process may not be feasible given time, personnel and funding constraints. Further, increasing the diversity in the responses considered “correct,” creates challenges with standardization across samples. If there is no single answer, how do we effectively conclude that athletes using a program have more adequate knowledge compared to another group? Though we do not have a solution, it is possible that leveraging digital technologies like artificial intelligence could help reduce some of the potential burden on research teams if sport nutrition measurement tools collected more nuanced responses.

## Conclusion

We believe tools that measure sport nutrition knowledge within research can be leveraged to be more practically useful for youth athletes. Though we do not have all the answers, we aim to spark greater discussion and collaborative efforts in the research community to define how we conceptualize and assess sport nutrition knowledge. Notably, we propose the establishment of sport end-user consensus on what practical knowledge entails, and how broader knowledge domains beyond nutrient needs can be included as a meaningful first step.
